# Clinical classification of cauda equina syndrome for proper treatment: A retrospective analysis of 39 patients

**DOI:** 10.3109/17453674.2010.483985

**Published:** 2010-05-21

**Authors:** Jiangang Shi, Lanshun Jia, Wen Yuan, GouDong Shi, Bin Ma, Bo Wang, JianFeng Wu

**Affiliations:** Orthopedics Department, ChangZheng Hospital, ShangHaiChina

## Abstract

**Background and purpose:**

Cauda equina syndrome (CES) is a severe complication of lumbar spinal disorders; it results from compression of the nerve roots of the cauda equina. The purpose of this study was to evaluate the clinical usefulness of a classification scheme of CES based on factors including clinical symptoms, imaging signs, and electrophysiological findings.

**Methods:**

The records of 39 patients with CES were divided into 4 groups based on clinical features as follows. Group 1 (preclinical): low back pain with only bulbocavernosus reflex and ischiocavernosus reflex abnormalities. Group 2 (early): saddle sensory disturbance and bilateral sciatica. Group 3 (middle): saddle sensory disturbance, bowel or bladder dysfunction, motor weakness of the lower extremity, and reduced sexual function. Group 4 (late): absence of saddle sensation and sexual function in addition to uncontrolled bowel function. The outcome including radiographic and electrophysiological findings was compared between groups.

**Results:**

The main clinical manifestations of CES included bilateral saddle sensory disturbance, and bowel, bladder, and sexual dysfunction. The clinical symptoms of patients with multiple-segment canal stenosis identified radiographically were more severe than those of patients with single-segment stenosis. BCR and ICR improved in groups 1 and 2 after surgery, but no change was noted for groups 3 and 4.

**Interpretation:**

We conclude that bilateral radiculopathy or sciatica are early stages of CES and indicate a high risk of development of advanced CES. Electrophysiological abnormalities and reduced saddle sensation are indices of early diagnosis. Patients at the preclinical and early stages have better functional recovery than patients in later stages after surgical decompression.

## Introduction

Cauda equina syndrome (CES) is a severe complication of lumbar spinal disorders; it results from compression of the nerve roots of the cauda equina. Patients typically present with a classic triad of saddle anesthesia, bowel and/or bladder dysfunction, and lower extremity weakness. As delay in diagnosis results in substantial morbidity, prompt diagnosis and therapy is essential (Gautschi et al. 2008).

There are many possible classifications of lumbar compression, based on location, disease type, or time of onset. It is unclear which scheme of classification of CES would be the most appropriate for clinical management. In this study, we sought to evaluate the clinical usefulness of a classification scheme of CES based on various factors including etiology, pathogenesis, clinical symptoms, imaging signs, and electrophysiological findings for the purpose of proper clinical management.

## Patients and methods

### Patient selection

In this retrospective study, the records of approximately 500 patients who had lumbar laminectomies performed for different reasons at our hospital from June 2000 through December 2006 were reviewed by 2 senior orthopedic physicians. From these, the records of 39 patients regarded to have CES, and who were operated on, were selected. The disorders that led to the need for surgery were: intervertebral disk protrusion (18), chiropractic manipulation for pre-existing disorders of the spine (9), over-traction caused by injury (3), lumbar spinal surgery (3), and lumbar trauma (6). All patients had sensory disturbances in the L2-3, L3-4, L4-5 and L5-S1 innervated areas. All patients had received decompressive laminectomy with an internal fixation device to stabilize the spine, and they were followed postoperatively for an average of 3 (2–6) years.

The criteria for decompressive laminectomy were compression caused by lumbar spinal canal narrowing and sensory disturbances. In order to ameliorate pressure on the dural sac, decompression laminectomy and incision of the spinal ganglion were performed as described previously (Bains et al. 2001). Internal stabilization was via lumbar pedical screw fixation. The time between diagnosis of CES and surgery was within 8 h for all patients.

Patients were divided into 4 groups as follows, based on clinical findings. Group 1: low-back pain with only bulbocavernosus reflex (BCR) and ischiocavernosus reflex (ICR) abnormalities and no typical symptoms of CES. Group 2: saddle sensory disturbance, numbness, and bilateral sciatica. Group 3: saddle sensory disturbance, numbness, bowel and/or bladder dysfunction, motor weakness of the lower extremities, and reduced sexual function. Group 4: absence of saddle sensation and sexual function, and uncontrolled bowel function. Clinical stages were defined as preclinical (group 1), early (group 2), middle (group 3), and late (group 4).

### Functional assessment

Bladder and bowel function were assessed according to clinical symptoms. Generally, dysfunction progressed from mild to more severe, i.e. from normal, to difficulty in defecation, to constipation, and to retention and incontinence (Nortvedt et al. 2007). Sexual function was classified into 4 categories as follows: grade 1, normal erection; grade 2, erection insufficiency, but able to achieve intercourse; grade 3, erection occurs, but unable to complete intercourse; grade 4, unable to achieve erection (Nogueira et al. 1990).

### Electrophysiology

Electrophysiological bulbocavernosus reflex (BCR) and ischiocavernosus reflex (ICR) examinations were performed on patients before and after surgery, as previously described (Ferdjallah et al.1996). Abnormalities were indicated by prolonged latencies. Specifically, BCR and ICR latency periods of > 33 ± 3.4 ms and > 34 ± 3.6 ms were considered abnormal, respectively. These cutoff values were determined by assessment of normal individuals (unpublished data).

### Imaging studies

Myelograms, CT, or MRI were employed for diagnostic purposes. 23 patients underwent myelography, 32 CT scans, and 12 MRI. Patients in whom CES had previously been diagnosed did not undergo myelography. The radiographic analyses were based on the radiologist's report in the records.

### Statistics

The results from the electrophysiological examination (BCR and ICR) before and after operation were compared by paired t-test. Continuous data are presented as mean (95% CI) while categorical data are represented as numbers (n). All parameter estimation was two-sided and was evaluated at the 0.05 level of significance. Statistical analyses were performed using SPSS version 15.0.

## Results

### Etiology and clinical features

We studied 39 patients (mean age 45 (26–85) years, 32 men). There were 8, 9, 9, and 13 patients in groups 1–4, respectively. Most patients had a similar clinical history beginning with low back pain and eventually progressing to incontinence of stool and urine. The time frame of progression could not be determined for all patients.

The initial manifestation of nerve root involvement in CES presented as sensory disturbance of the saddle region, and there was a positive correlation between the severity of the sensory disturbance and the number of nerve roots involved. In our 39 patients, most of sensory disturbances were found in the L3-4 and L5-S1 innervated areas. Causes of compression included herniated discs, spinal stenosis, and hematoma. While all the causes of stenosis were noted in patients in groups 3 and 4, patients in group 1 were only found to have stenosis secondary to herniated discs. A representative image of a patient with CES in group 4 is shown in the Figure.

**Figure:**
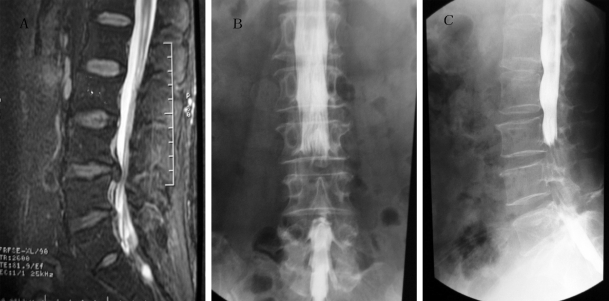
A 38-year-old male from group 4. The patient experienced bowel incontinence and inability to achieve erection after chiropractic spinal manipulation. The dural sac and cauda equina were compressed at multiple levels, including L3-4, L4-5, and L5-S1. Myelography revealed severe dural sac compression with bilateral involvement. A. Lumbar MRI sagittal radiograph showing the L4-5 herniated disk in the narrow area. B. Frontal radiograph of the lumbar spinal canal showing a non-filling narrow area corresponding to the L4-5 herniated disk. C. Lateral radiograph of the lumbar spinal canal showing a narrow non-filling area corresponding to the L4-5 herniated disk.

### Image analysis

Myelograms taken before and after the onset of CES typically revealed aggravation of spinal canal obstruction or even complete obstruction. CT scanning showed spinal stenosis and intervertebral disc degeneration and herniation. MRI revealed multilevel compression of the dural sac. Stenosis level and range, compression location, and cause of compression were compared for the 4 groups (Table 1). Stenosis at the level of L3-4, L4-L5, or L5-S1 was detected in patients in all groups; however, stenosis at the level of L2-4 was only seen in group 4. The range of stenosis correlated with the severity of CES. Patients in group 1 had stenosis at 1 or 2 segments, whereas group 4 where patients were found to have stenosis at 2–4 segments. Patients in groups 3 and 4 had single or bilateral nerve root compression whereas patients in group 1 only had dural sac compression.

**Table 1.** Imaging findings preoperatively. Data presented are numbers of patients

	Group 1 (n = 8)	Group 2 (n = 9)	Group 3 (n = 9)	Group 4 (n = 13)
Stenosis level				
L2-4	0	0	0	7
L3-4	5	2	4	0
L4-5	1	1	0	0
L5-S1	1	2	1	0
L4-5 and L5-S1	1	4	4	6
Range ^**a**^				
1	5	2	0	0
2	3	4	4	3
3	0	3	5	6
4	0	0	0	4
Compression				
Dural sac	8	4	0	3
Single nerve root	0	4	3	2
Bilateral nerve root	0	1	6	8
Cause of compression				
Discs	5	4	4	5
Spinal stenosis	0	3	3	6
Hematoma	0	0	2	1
None	3	2	0	1
Group 1 patients had low back pain with only BCR (bulbocavernosus reflex) and ICR (ischiocavernosus reflex) abnormalities and no typical symptoms of CES.				
Group 2 patients had slight saddle sensory disturbances and bilateral sciatica.				
Group 3 patients had severe saddle sensory disturbances, bowel and/or bladder dysfunction, motor weakness of the lower extremities and reduced sexual function.				
Group 4 patients had no saddle sensation, no sexual function, and uncontrolled bowel function.				

^**a**^ 1 represents one segment of stenosis, 2 represent two segments of stenosis, etc.				

### Functional assessment

Pre- and post-surgical bowel and bladder function was compared. Immediately after surgery, bowel and bladder function was improved in patients in groups 2 and 3 (Table 2). No changes were noted for patients in group 4 (Table 2). Notably, 1 more patient in group 3 and 1 in group 4 experienced fecal retention after surgery rather than before, and fecal retention was chronic in these patients. Patients in group 1 had no clinical abnormalities preoperatively. Likewise, sexual function was improved in groups 2 and 3, but no changes were noted in group 4 (Table 3). With regard to electrophysiological examination, BCR and ICR were improved in groups 1 and 2 after surgery; however, no change was noted for patients in groups 3 and 4 (Table 4).

**Table 2.** Bowel and bladder function pre- and post-surgery. Data presented are numbers of patients (before / after)

	Group 1 (n = 8)	Group 2 (n = 9)	Group 3 (n = 9)	Group 4 (n = 13)
Normal	8 / 8	7 / 9	2 / 4	0 / 0
Mild dysfunction	0 / 0	2 / 0	5 / 2	1 / 1
Fecal retention	0 / 0	0 / 0	2 / 3	4 / 5
Urinary retention	0 / 0	0 / 0	0 / 0	8 / 7

For group classifications, see Table 1.

**Table 3.** Sexual function pre- and post-surgery. Data presented are numbers of patients (before / after)

	Group 1 (n = 8)	Group 2 (n = 9)	Group 3 (n = 9)	Group 4 (n = 13)
Grade 1 ^**a**^	8 / 8	7 / 9	4 / 6	0 / 0
Grade 2	0 / 0	2 / 0	2 / 1	2 / 2
Grade 3	0 / 0	0 / 0	2 / 1	3 / 5
Grade 4	0 / 0	0 / 0	1 / 1	8 / 6

For group classifications, see Table 1.

^**a**^ Grade 1 indicates normal arousal.

**Table 4.** Results of electrophysiological examinations (BCR and ICR) pre- and post-surgery. Data are presented as mean (95% CI).

	Group 1 (n = 8)	Group 2 (n = 9)	Group 3 (n = 9)	Group 4 (n = 13)
BCR (ms)				
pre-surgery	47 (43–51)	48 (42–54)	55 (50–60)	58 (54–62)
post-surgery	34 (3–37) ^**a**^	41 (37–44) ^**a**^	51 (48–54)	57 (56–59)
ICR (ms)				
pre-surgery	44 (40–48)	46 (41–51)	51 (49–54)	56 (53–60)
post-surgery	33 (31–35) ^**a**^	38 (36–41) ^**a**^	50 (48–53)	56 (55 –56)

For group classifications, see Table 1.

^**a**^ p < 0.05 (paired t-test).

## Discussion

CES refers to impairment of the cauda equina and clinically it may manifest as low back pain, saddle anesthesia, bilateral sciatica, motor weakness of the lower extremities, paraplegia, and bowel, bladder, or sexual dysfunction (Byrne 1993). Although this definition may include patients without CES, this concept of CES can allow the clinician to identify patients who have a high risk of developing “full-blown CES” (Anderson 1997, Elderfield 1999).

The common symptoms of early CES include low back pain and radicular leg pain. Generally, low back pain results from irritation of the soft tissue close to the lumbar spinal canal and bony elements (Borenstein 1994). Some cases of early CES may present with only unilateral or bilateral sciatica. Notably, in our study we found that some patients in group 1 (defined as preclinical) only experienced low back pain with BCR and ICR abnormalities. It has been shown that electrophysiological recordings (BCR and ICR) can provide evidence of lumbar spinal stenosis when neurological examination does not show any specific sensory-motor deficit (Egli et al. 2007). We found the same by the radiological identification of stenosis in patients with abnormal electrophysiological findings, but no specific sensory-motor deficits on examination.

In addition, our results demonstrated that surgery could help patients with slight saddle sensory disturbance and bilateral sciatica (patients in group 2), or severe saddle sensory disturbance, bowel or bladder dysfunction, motor weakness of the lower extremities and reduced sexual performance (patients in group 3). We postulate that patients in group 1 did not directly benefit from surgery (but it probably prevented further progression) because in the preclinical stage, compression was not severe enough to be altered by surgical correction. Conversely, patients with extensive nerve damage from severe and prolonged compression (group 4) were not helped by surgical intervention.

As the development of CES follows a natural process, we classified patients as being preclinical, early-, middle-, or late-stage (groups 1–4) based on clinical findings. Our results showed that the physiological dysfunction (such as bladder or bowel dysfunction) was in accordance with the severity of CES seen among the groups. Based on our findings, it appears that different stages of CES manifest with different clinical features and should be treated differently. Surgical goals are to relieve the local compression as soon as possible, to alleviate or prevent the initiation of the tissue damage caused by local compression, to reduce the high pressure in the posterior ganglia of the nerve roots, to alleviate ischemia and hypoxia, to reduce the degeneration and death of the sensory neurons in the ganglia, and at the same time reduce the retrograde degeneration and inhibit the apoptosis of the sacral anterior horn cells. For patients with preclinical CES, simple fenestration can be performed to relieve compression. For early-and middle-stage patients, nerve root decompression should be performed as early as possible. While surgical treatment is less effective for late-stage patients, nonoperative treatment of late-stage patients delays appropriate management and most likely leads to poorer outcomes (Lorenz 1985). Expanded decompression should be considered for patients with intermittent claudication before the onset of CES.

In addition to impairment of the conus medullaris, nerve fiber and posterior ganglion damage affects the integrity of the reflex arch, resulting in dysfunction of defecation, urination, and sexual performance. The closer the pressure to the conus, the more severe the impairment to the cauda equina and the greater number of anterior horn cells involved (Sato and Kikuchi 1997). Disintegration of the bilateral reflex arches is believed to be the pathogenesis of cauda equina injury. Thus, bilateral radiculopathy may be the critical symptom that indicates a high risk of development of CES.

In summary, CES can be classified into 4 stages in clinical practice—preclinical, early, middle, and late—based on clinical symptoms and physiological dysfunction. This clinical classification provides a reference for diagnosis of CES at various stages, and may lead to better outcomes by establishing an appropriate therapeutic strategy earlier in the course of the disease.
